# Simultaneous and enantiospecific quantification of primaquine and carboxyprimaquine in human plasma using liquid chromatography-tandem mass spectrometry

**DOI:** 10.1186/s12936-022-04191-w

**Published:** 2022-06-03

**Authors:** Warunee Hanpithakpong, Nicholas P. J. Day, Nicholas J. White, Joel Tarning

**Affiliations:** 1 Department of Clinical Pharmacology, Mahidol Oxford Tropical Medicine Research Unit, Faculty of Tropical Medicine, Mahidol University, Bangkok, Thailand; 2 Centre for Tropical Medicine and Global Health, Nuffield Department of Medicine, University of Oxford, Oxford, UK

**Keywords:** Primaquine, Enantiomeric separation, Malaria, LC–MS/MS validation, Antimalarial drugs

## Abstract

**Background:**

The enantiomers of the 8-aminoquinoline anti-malarial primaquine have different pharmacological properties. Development of an analytical method for simultaneous quantification of the enantiomers of primaquine and its metabolite, carboxyprimaquine, will support clinical pharmacometric assessments.

**Methods:**

A simple and sensitive method consisting of liquid chromatography coupled with tandem mass spectrometry (LC–MS/MS) was developed for simultaneous and enantiospecific determination of primaquine and its metabolite, carboxyprimaquine, in human plasma. Stable isotopes were used as internal standards to compensate for potential interference and matrix effects. Plasma samples (100 µL) were precipitated with 1% formic acid in acetonitrile followed by phospholipid removal solid phase extraction. Primaquine and carboxyprimaquine enantiomers were separated on a Chiralcel OD-3R (150 mm × 4.6 mm; I.D. 3 μm) column using a LC gradient mode. For separation of racemic primaquine and carboxyprimaquine, the LC method was modified and validated using a reverse phase column (Hypersil Gold 100 mm × 4.6 mm; I.D. 3 µm) and a mobile phase composed of 10 mM ammonium acetate buffer, pH 3.5 and acetonitrile in the isocratic mode. Method validation was performed according to regulatory guidelines.

**Results:**

The calibration range was set to 0.571–260 ng/mL and 2.44–2,500 ng/mL for primaquine and carboxyprimaquine enantiomers, respectively, resulting in a correlation coefficient (r^2^) ≥ 0.0998 for all calibration curves. The intra- and inter-day assay precisions were < 10% and the accuracy was between 94.7 to 103% for all enantiomers of primaquine and carboxyprimaquine. The enantiospecific method was also modified and validated to quantify racemic primaquine and carboxyprimaquine, reducing the total run time from 30 to 8 min. The inter-, intra-day assay precision of the racemic quantification method was < 15%. The absolute recoveries of primaquine and carboxyprimaquine were between 70 and 80%. Stability was demonstrated for up to 2 years in − 80 °C. Both the enantiomeric and racemic LC–MS/MS methods were successfully implemented in pharmacokinetic studies in healthy volunteers.

**Conclusions:**

Simple, sensitive and accurate LC–MS/MS methods for the quantification of enantiomeric and racemic primaquine and carboxyprimaquine in human plasma were validated successfully and implemented in clinical routine drug analysis.

## Background

**Fig. 1:**
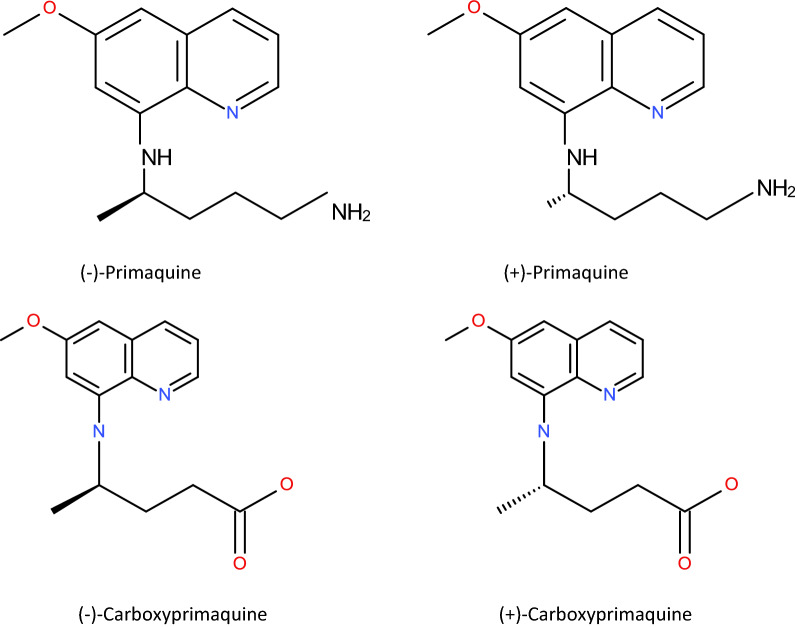
Molecular structure of (−)-primaquine, ( +)-primaquine, (−)-carboxyprimaquine and ( +)-carboxyprimaquine

Primaquine (PRQ) is an 8-aminoquinoline anti-malarial drug (Fig. 1) used for the radical cure of relapsing malaria, as a gametocytocide in falciparum malaria and in malaria chemoprophylaxis. It is a chiral compound, marketed for clinical use as a racemate and usually administered as the phosphate salt [1]. The major adverse effect of primaquine is oxidant hemolysis, especially in glucose-6-phosphate dehydrogenase (G6PD) deficient patients [2–4].

Stereo-selective in vivo and in vitro studies have shown that the individual primaquine enantiomers have different pharmacological and toxicological properties [5–9]. Schmidt et al. reported that the curative anti-malarial activity of racemic primaquine and its individual enantiomers was identical when studied in rhesus monkeys infected with *Plasmodium cynomolgi* (an animal model of vivax malaria), but that (−)-primaquine was at least twice as toxic as ( +)-primaquine [6]. This was supported by in vitro studies demonstrating that (−)-primaquine produced a significantly greater effect in reducing glutathione and increasing methemoglobin levels in normal and G6PD red blood cells compared to ( +)-primaquine [7]. However, Nanayakkara and colleagues presented conflicting results in rodent models and beagle dogs, where ( +)-primaquine was found to be both more efficacious and more hemotoxic compared with (-)-primaquine [10]. Similarly, in the *P. cynomolgi* challenge model in rhesus macaques there was a greater rise in methemoglobin after receiving ( +)-primaquine compared with (−)-primaquine. In the same model, chloroquine in combination with (−)-primaquine was more effective in preventing *P. cynomolgi* relapse compared with ( +)-primaquine [9].

When racemic primaquine was administered to rats, a small fraction of the dose was excreted unchanged in the urine as ( +)-primaquine with negligible amounts of (−)-primaquine [5]. Primaquine is metabolized by monoamine oxidase to its major circulating metabolite, carboxyprimaquine, believed to be inactive [11, 12]. Primaquine requires bioactivation for its biological effects. Pybus et al. demonstrated that metabolism of primaquine by CYP2D6 is essential for radical cure of *Plasmodium vivax* malaria [13]. In vitro CYP2D6-mediated metabolism studies demonstrated that 2- and 5-hydroxyprimaquine were preferentially generated from ( +)-primaquine, while 3- and 4-hydroxyprimaquine was preferentially generated from (−)-primaquine [1]. Furthermore, In in vitro studies using human hepatocytes showed two major routes of metabolism; oxidative-deamination of the sidechain terminal amine and hydroxylation of the quinoline moiety [14]. The major deaminated metabolite, carboxyprimaquine, was preferentially generated from (−)-primaquine while the hydroxylated products were preferentially formed from ( +)-primaquine. However, only trace amounts of the hydroxylated metabolites were detected with hepatocyte incubations, all exclusively generated from ( +)-primaquine. The contributions to anti-relapse activities of these metabolites are unknown. The first enantiospecific pharmacokinetic study of primaquine in healthy human volunteers after racemic primaquine administration showed a higher exposure to ( +)-primaquine compared to (−)-primaquine. Only (−)-carboxyprimaquine was detected in plasma using LC/MS-TOF [15].

Different methods using LC-UV [12, 16–19], LC-EC [20] and LC–MS techniques [18, 21, 22] for the quantification of racemic primaquine and/or carboxyprimaquine have been described in the literature. To date, only one method describes enantiospecific separation and quantification of primaquine and carboxyprimaquine, using LC–MS-TOF [23]. However, the authors report a total run time of almost 50 min per sample, which makes this method difficult to implement in routine drug quantification of large clinical studies.

The primary aim of this study was to develop and validate a sensitive, accurate and rapid bioanalytical method for enantiospecific separation and detection of primaquine and carboxyprimaquine, suitable for high-throughput use in clinical studies. A secondary aim was to modify and optimize the analytical method to enable a substantially shorter analysis time when quantifying racemic primaquine and carboxyprimaquine.

## Methods

### Chemicals & reagents

Racemic primaquine (PRQ) and carboxyprimaquine (CPRQ) were provided by the WorldWide Antimalarial Resistance Network (WWARN) [24]. Enantiomeric primaquine (( ±)-PRQ; Fig. 1), stable isotope-labelled primaquine (( ±)-PRQ-D3) and carboxyprimaquine (( ±)-CPRQ-D3) were kindly provided by Prof Larry Walker, National Centre for Natural Products Research, University of Mississippi, USA. LC–MS grade acetonitrile, methanol and water were obtained from J.T. Baker (Phillipsburg, NJ, USA). Dimethyl sulphoxide (AR grade), ammonium acetate and ammonium formate (LC–MS grade) were obtained from Fluka/Sigma-Aldrich (Sigma-Aldrich, St. Louis, MO, USA). Formic acid (LC–MS grade) was obtained from Merck (Merck Darmstadt, Germany).

### Instrumentation, separation and detection

Sample preparation and solid-phase extraction was performed on an automated Freedom Evo 200 platform (TECAN, Männedorf, Switzerland). The LC system was an Agilent 1200 system consisting of a binary LC pump, a vacuum degasser, a temperature-controlled micro well plate auto-sampler set at 4 °C and a column compartment set at 20 °C (Agilent technologies, Santa Clara, CA, USA). The mass spectrometry equipment was an API 5000 triple quadrupole system (Applied Biosystems/MDS Sciex, Foster City, CA, USA), with a TurboV Ionization Source (TIS) interface operated in the positive ion mode. Data acquisition was performed using Analyst 1.5 (Applied Biosystems/MDS Sciex, Foster City, CA, USA).

PRQ, PRQ-D3, CPRQ and CPRQ-D3 enantiomers were separated on a Chiralcel OD-3R column (150 mm × 4.6 mm, I.D. 3 μm; Chiral Technologies Inc., West Chester, PA, USA), protected by a Chiralcel OD-3R guard column (4 mm × 10 mm, I.D. 3 μm), at a flow rate of 1.0 mL/min. Mobile phase A contained 20 mM ammonium formate:acetonitrile, 75:25 v/v with 0.1% formic acid, and mobile phase B contained methanol:acetonitrile (75:25, v/v). The following gradient program was employed for separation of enantiomeric PRQ and CPRQ and to remove phospholipid residues from the column: 0–15 min (100% mobile phase A), 15–16.0 min (100% mobile phase A to 100% mobile phase B), 16.0–20.8 min (100% mobile phase B), 20.8–21 min (100% mobile phase B to 100% mobile phase A), and 21.0–26.5 min (100% mobile phase A).

The MS/MS conditions were optimized by infusing ( ±)-PRQ (10 ng/mL) and ( ±)-CPRQ (20 ng/mL) at 10 μL/min, using a Harvard infusion pump connected directly to the MS. Additional MS/MS tuning was performed by continuous infusion of ( ±)-PRQ (25 ng/mL) and ( ±)-CPRQ (50 ng/mL) at a flow rate of 20 μL/min via a “T”-connector into the post-column mobile phase at a flow rate of 1.0 mL/min. TIS temperature was maintained at 700 °C and the TIS voltage was set to 4500 V. The curtain gas was set to 30 psi and the ion source gas 1 (GS1) and ion source gas 2 (GS2) at 50 and 60 psi, respectively. The CAD gas in the collision cell was set to 5 psi. Quantification was performed using multiple reaction monitoring (MRM) for the transitions m/z 260–175 and m/z 263–86 for PRQ and PRQ-D3, respectively, and m/z 275–175 and m/z 278–178 for CPRQ and CPRQ-D3, respectively. The declustering potential (DP) was set to 60 V for all analytes and stable isotope-labelled internal standards.

### Preparation of standards and quality control samples

Stock solutions of ( ±)-PRQ (0.5 mg/mL) and ( ±)-PRQ-D3 (1 mg/mL) were prepared in acetonitrile:water (50:50, v/v) and stock solutions of ( ±)-CPRQ (1 mg/mL) and ( ±)-CPRQ-D3 (1 mg/mL) were prepared in dimethylsulfoxide:methanol (1:3, v/v). Working solutions were prepared by serial dilutions in acetonitrile:water (50:50, v/v). Working solutions of ( ±)-PRQ-D3 and ( ±)-CPRQ-D3 (5 μg/mL and 25 μg/mL, respectively) were stored in 100 µL aliquots at − 80 °C until analysis. Calibration standards and quality control (QC) samples were prepared by adding working solution to human EDTA plasma (sourced from the Healthy volunteer ward at the Hospital for Tropical Medicine). The total content of working solution was less or equal to 2% of the total plasma volume in all cases except for the over curve dilution sample where it was 4%. Six calibration standards, excluding zero concentration, were prepared at 0.571–260 ng/mL for each enantiomer of PRQ and 2.44–2,500 ng/mL for each enantiomer of CPRQ, and stored at − 80 °C until analysis. Quality control samples for accuracy and precision of each enantiomer of PRQ and CPRQ were prepared at 3 × lower limit of quantification (LLOQ), mid-range and upper range (i.e. 1.46, 16.8 and 195 ng/mL for PRQ, and 7.32, 117 and 1,875 ng/mL for CPRQ). All QC samples were stored at − 80 °C until analysis.

### Analytical procedure

Plasma samples (100 μL) were aliquoted onto a 1 mL 96-wellplate. Solid phase extraction was performed by adding precipitation solution (300 μL of 1% formic acid in acetonitrile) containing 5 ng/mL of ( ±)-PRQ-D3 and 50 ng/mL of ( ±)-CPRQ-D3. The 96-well plate was covered with a methanol-washed seal mat and mixed on a Mixmate (Eppendorf, Germany) at 1000 rpm for 2 min followed by centrifugation at 1100*g* for 5 min. 200 μL of supernatant was loaded directly onto the phospholipid removal SPE (solid-phase extraction) plate (HybrideSPE-Phospholipid, Sulpelco, USA) and passed through by continuously increasing the vacuum. The eluent was diluted with 200 μL of dilution solution, containing methanol and 20 mM ammonium formate (75:25, v/v). The elution sample plate was covered with a methanol-washed Nunc pre-slit seal mat, mixed on a Mixmate at 900 rpm for 2 min and centrifuged at 1100 g for 2 min. A total volume of 5 μL was injected into the LC–MS/MS system.

### Validation

The enantiospecific method was subjected to a full validation according to the US-FDA guidelines, including assessment of linearity, precision, accuracy, short-term/long-term stability and matrix effects [25].

### Calibration and linearity

The calibration curve was set to 0.571–260 ng/mL and 2.44–2,500 ng/mL, for each enantiomer of PRQ and CPRQ, respectively. These calibration curves covered the ranges of expected therapeutic drug concentrations in plasma, based on observations following a single oral dose of 30 mg (base) primaquine phosphate in volunteers [26, 27]. Each calibration level was run in duplicate except at the lower limit of quantification (LLOQ), which was run in five replicates during the four days of validation. Linear and quadratic regression models of the calibration curve response (peak area ratios of ( ±)-PRQ/ ( ±)-PRQ-D3 and ( ±)-CPRQ/( ±)-CRQ-D3) with and without weighting (1/x and 1/x^2^) were evaluated for each calibration curve. The optimal regression model was chosen on the basis of back-calculated concentrations of calibration standards (i.e., relative bias of back-calculated concentrations compared to nominal values) as well as the accuracy of predicted QC samples. Selection of the best performing regression model was based on the ranking approach suggested by Singtoroj and colleagues [28].

### Precision and accuracy

Precision and accuracy were assessed by daily analysis of five replicates of QC samples (three concentration levels), LLOQ samples and upper limit of quantification (ULOQ) samples over the four days of validation. Five replicates of over-curve dilution were assessed by a five-fold dilution of spiked standards at 1038 and 10,000 ng/mL of ( ±)-PRQ and ( ±)-CPRQ, respectively. Intra-, inter- and total-assay precision of QC samples, LLOQ, ULOQ and over curve dilution samples were calculated using analysis of variance (ANOVA). The acceptance criteria for accuracy and precision was ± 15% except for the LLOQ, which was ± 20% [25].

### Stability and carry-over

Short-term and long-term stability were evaluated in five replicates of QC samples at low and high concentrations. Short-term stability of ( ±)-PRQ and ( ±)-CPRQ in human EDTA plasma was evaluated during three freeze/thaw cycles, at ambient temperature and at 4 °C for 48 h. Bench-top stability was evaluated for thawed samples ready for extraction (2 h at ambient temperature) and for extracted samples ready for injection (60 h at 4 °C in autosampler). Long-term stability was evaluated in EDTA plasma stored at − 80 °C for 6 months, 1 and 2 years. Potential carry-over effects were evaluated by injection of three extracted blank samples directly after injection of the highest concentration in the calibration curve.

### Matrix effects, absolute recovery, and selectivity

Two sets of blank plasma from six different donors were used for evaluation of matrix effects and recovery. The first set of blank samples was spiked to low and high QC concentrations and extracted following the procedure described above (*P*_*pre-spiked*_) in the Analytical procedure section. The second set of blank samples was extracted following the Analytical procedure described above except the eluent was diluted with dilution solution, containing PRQ and CPRQ, to low and high QC concentrations (*P*_*post-spiked*_). Reference solution was also spiked to low and high QC concentrations (*P*_*neat-solution*_) in mobile phase A. All samples were injected and quantified using the developed method, and assessed by calculating absolute recovery (Eq. 1), matrix factor for both analytes and internal standards (Eq. 2) and normalized matrix effect (Eq. 3) [29, 30].

1 $$Absolute\;recovery\left( \% \right) = \frac{{Average\;response\;P_{pre - spiked} }}{{Average\;response\;P_{post - spiked} }} \times 100$$

2 $$Matrix\;factor\; = \;\frac{{Average\;response\;P_{post - spiked} }}{{Average\;response\;P_{{neat{ }solution}} }}$$

3 $$Normalized\;matrix\;effect\; = \;\frac{{Matrix\;factor_{analyte} }}{{Matrix\;factor_{internal\;standard} }}$$

No substantial matrix effect was assumed if the calculated matrix factor was between 0.85 and 1.15 (ion suppression; < 0.85 or > 1.15 ion enhancement) [29]. The internal standard is expected to compensate for a potential matrix effect if the calculated normalized matrix effect was between 0.85 and 1.15. Graphical evaluation of the matrix effect was also performed through post-column infusion experiments as described elsewhere [31, 32]. Briefly, ( ±)-PRQ (25 ng/mL) and ( ±)-CPRQ (50 ng/mL) solutions were infused continuously at 20 μL/min into the mass spectrometer while extracted blank plasma samples were injected. Potential ion suppression or enhancement were investigated by evaluating the PRQ and CPRQ intensity at the retention times of PRQ, CPRQ and the internal standard enantiomers.

Selectivity was evaluated by trace analysis of the blank plasma extracted from six different donors. All blank sources should produce a response less than or equal to 20% of the response of the lowest standard (i.e., LLOQ). Interference by potentially co-administered anti-malarial drugs (i.e., piperaquine, dihydroartemisinin, chloroquine and pyronaridine) was also evaluated. Possibly interfering drugs were assessed by continuous post-column infusion of ( ±)-PRQ (25 ng/mL) and ( ±)-CPRQ (50 ng/mL), and injection of mobile phase A spiked with concomitant anti-malarial drugs. Potential interference was evaluated visually by enhancement/suppression of ( ±)-PRQ and ( ±)-CPRQ signals at the retention times of PRQ, CPRQ and internal standard enantiomers.

### Partial validation of racemic primaquine quantification

The separation method was modified and subject to a partial validation according to the US-FDA bioanalytical method validation guidelines [25]. Racemic PRQ, PRQ-D3, CPRQ and CPRQ-D3 were separated on a Hypersil gold column (100 mm × 4.6 mm, I.D. 3 μm; Thermo Fisher Scientific, USA), protected by a Hypersil gold guard column (2.1 × 10 mm, I.D. 5 μm) at a flow rate of 0.5 μL/min and a total run time of 8 min. The isocratic mobile phase consisted of 10 mM ammonium acetate pH 3.5: acetonitrile, 50:50 v/v. Sample preparation and Q2-MS settings were identical to that described above for enantiomeric PRQ and CPRQ. However, front-end MS parameters were slightly modified for optimal performance; TIS temperature was maintained at 500 °C and the ion source gas (GS1) was set to 60 psi.

Precision and accuracy were assessed by daily analysis of five replicates of LLOQ, QC and ULOQ samples over four days. The validation range of racemic PRQ and CPRQ was 1.14–519 and 4.88–5000 ng/mL, respectively. Five replicates of over-curve dilution were assessed by a five-fold dilution of spiked standards at 1038 ng/mL and 10,000 ng/mL of PRQ and CPRQ, respectively. Matrix effects and regression models were evaluated as previously described for the enantiomeric quantification method.

### Clinical applicability

The developed bioanalytical method for quantification of enantiomeric and racemic PRQ and CPRQ was successfully implemented in three clinical drug-drug interaction studies in Thai volunteers receiving a single oral dose of primaquine phosphate (30 mg) with and without the commonly used anti-malarial drugs chloroquine [33], artesunate-pyronaridine [34] and dihydroartemisinin-piperaquine [35]. A total of 800 plasma samples from two of the above studies were analysed using the developed enantiomeric quantification method for ( ±)-PRQ and ( ±)-CPRQ [34, 35], and a total of 500 samples were analysed using the developed quantification method for racemic PRQ and CPRQ [33]. Venous blood samples were collected frequently into fluoride-oxalate blood collection tubes and centrifuged to obtain plasma. The developed methods were implemented using a high-throughput LC–MS/MS system for enantiomeric and racemic methods in the 96-well format as described above. Each batch of drug measurements (96-well plate) was accepted based on the performance of triplicates of QC samples at three concentrations and incurred sample reanalysis (ISR) of 10% of samples.

## Results and discussion

The aim of this investigation was to develop a simple, sensitive and high-throughput enantiomeric quantification method of the anti-malarial drug PRQ and its main metabolite, CPRQ. An enantiomeric separation and quantification method were successfully developed, validated and implemented for the pharmacokinetic analysis of clinical samples. This method demonstrated improved sensitivity and reduced runtime compared to previously described methods. The complexity of the enantiomeric method can be reduced in racemic quantification of PRQ and CPRQ while maintaining a high sensitivity. The total run time was reduced to only 8 min for the racemic method, enabling a high-throughput automated routine analysis of clinical pharmacokinetic samples.

### Chromatography and quantification

**Fig. 2:**
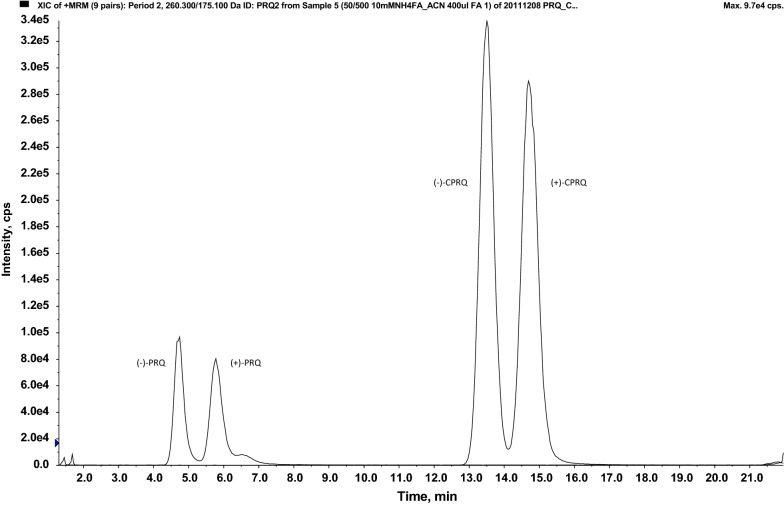
Retention time of enantiomeric primaquine and carboxyprimaquine on the Chiralcel OD-3R column (150 mm × 4.6 mm; I.D. 3 μm) at flow rate 1.0 mL/min, using a gradient program consisting of mobile phase A (20 mM ammonium formate:acetonitrile; 75:25, v/v with 0.1% formic acid) and mobile phase B (methanol:acetonitrile; 75:25, v/v)

The developed LC gradient method allowed for complete separation of PRQ and CPRQ enantiomers and internal standards in less than 30 min, including washout and re-equilibration (Fig. 2). The gradient program ended with 100% organic solvent in order to elute phospholipids that might otherwise accumulate on the column and reduce column performance over the time and cause matrix effects [29, 30, 36].

Bonato and colleagues reported the first enantiomeric separation of PRQ and CPRQ using chiral columns including Chiralcel OD-H, Chiralcel OD-R and Chiralpak AGP [37], while Avula and colleagues reported the first validated method for enantiomeric quantification of PRQ and CPRQ using a Chiralcel OD-R column [23]. However, this method required a long analytical run time of 50 min per sample.

Several different types of chiral columns (e.g., Chiralcel OD-R, Chiralpak-AGP, Chiralpak-IB) were evaluated, which all resulted in long run times or unresolved separation between enantiomers [23, 37]. The Chiralcel OD-3R (150 mm × 4.6 mm; I.D. 3 μm particle size) column enabled an improved run time, while retaining adequate sensitivity and peak resolution. Chiralcel OD-3R is a reverse phase chiral column coated with 3 μm silica-gel. This column has a similar stationary phase to the Chiralcel OD-R (250 mm × 4.6 mm; I.D. 10 μm particle size) used previously by Avula [23]. However, the smaller particle size in the Chiralcel OD-3R column, allowed faster separation and higher peak resolution. Even so, separation of PRQ and CPRQ enantiomers were not satisfactory without acidification of the mobile phase. Therefore, the amount of formic acid, ionic strength of the ammonium formate buffer and organic solvent were optimized. It has been suggested that retention and enantio-selectivity of amphipathic, PRQ and amphoteric, CPRQ, compounds with reverse phase chiral columns depends on hydrophobic interactions, hydrogen binding, ionic bonding and ion pairing interactions [23, 37]. Thus, retention of PRQ was increased by increasing the pH and reducing the amount of acetonitrile in the mobile phase. However, retention of CPRQ was increased by decreasing the mobile phase pH as a result of ionization of the carboxylic moiety. The optimal ionic strength of the mobile phase buffer (range: 5–20 mM) and the acidic conditions (range: 0.1–1%) were optimized for improved peak resolution and shape. Finally, 20 mM ammonium formate:acetonitrile (75:25 v/v) with 0.1% formic acid was selected, using a gradient program ending with 100% organic solvent (methanol–acetonitrile) to elute accumulated phospholipids and therefore extend the column life and avoid matrix carry over to the subsequent sample. (−)-PRQ, ( +)-PRQ, (−)-CPRQ and ( +)-CPRQ had retention times of 4.73, 5.71, 13.5 and 14.7 min, respectively.

The MS/MS fragments were selected based on the most abundant transition signals, compound purity, selectivity, sensitivity (as measured by signal-to-noise ratio) and analyte contribution. Quantification of all analytes was performed using MRM transitions of m/z 260.3 > 175.1 and 263.3 > 86.1 for PRQ and PRQ-D3, respectively, and 275.2 > 175.1 and 278.4 > 178.1 for CPRQ and CPRQ-D3, respectively. The collision energy was set to 30 V for all compounds. In order to achieve successful quantification of the enantiomers when connected to the LC-column, the ESI + tuning parameters were optimized for the protonated precursor and product ions of the analytes and internal standards. The developed detection method resulted in an unbiased and robust method with high sensitivity.

### Sample preparation

Sample preparation and drug extraction was achieved by protein precipitation followed by phospholipid removal SPE, enabling automation and high-throughput analyses in the 96-well plate format. This extraction technique provided a clean sample with no residual interference, and it can be automated easily to enable high-throughput analyses in the 96-well plate format. The disadvantage of solid-phase extraction is a higher per sample cost compared to simplified extraction protocols such as direct protein precipitation.

Previously published methods have used liquid–liquid extraction, requiring separate processes to extract PRQ and CPRQ from clinical plasma samples [12, 16, 18, 38]. Such an extraction approach is laborious and time consuming, especially when parent and metabolite compounds require separate processing. Laborious and manual methods are difficult to automate for a high-throughput approach when analysing pharmacokinetic clinical trial samples. Furthermore, larger sample volumes are commonly needed, which is not always practically or ethically achievable in malaria field studies.

Direct protein precipitation with methanol or acetonitrile has also been reported in the literature [23, 39, 40]. Direct protein precipitation is the most commonly used extraction technique and can be implemented in a high-throughput setting. However, residues from this simple extraction technique can result in substantial matrix effects, especially when complex separation of several compounds and internal standards are needed [31]. One previously published method utilized solid-phase extraction (Oasis HLB cartridges) resulting in a robust extraction technique and a sensitive method with an LLOQ of 2 ng/mL [22]. However, the calculated relative matrix effect was reported to be close to 15% with a relatively high variation between batches. Thus, this particular extraction assay might suffer from variable matrix effects affecting the precision and accuracy of the assay [31].

Protein precipitation followed by phospholipid removal SPE was developed using methanol or acetonitrile in 0.1–2% acetic or formic acid conditions. During method development, the efficiency of the extraction (i.e., recovery) and the residual phospholipids was compared between direct protein precipitation with and without phospholipid removal SPE. Direct protein precipitation without phospholipid removal SPE showed better recovery but more residual phospholipids. Direct protein precipitation with phospholipid removal SPE showed acceptable recovery (70–80%) and less residual phospholipids. The impact of the small amounts of phospholipids, still found after using phospholipid removal SPE, could be minimized by the gradient LC program ending with 100% organic solvent to waste before next sample injection.

## Validation

### Calibration curve and carry-over.

The calibration curve was constructed using six calibration standards, not including zero. The calibration range for each enantiomer of PRQ (0.571–260 ng/mL) and CPRQ (2.44–2500 ng/mL) quantification was set to cover expected peak concentrations associated with the 14 days radical cure regimen [27, 41–43]. Linear and quadratic regression models with 1/x^2^ weighting generated similar results with respect to accuracy and precision, and could be used interchangeably. However, the accuracy was somewhat higher for the quadratic regression model for ULOQ samples compared to the linear regression model (85–90% versus 95–105%). Therefore, the quadratic regression model with 1/x^2^ weighting was selected for the final assay. All other regression models tried showed different degrees of bias.

None of the blank samples produced any trace signals of PRQ, CPRQ, PRQ-D3, or CPRQ-D3 enantiomers after three replicate injections of the highest concentration of the calibration curve. Thus, no carry-over effects were seen, suggesting that the gradient program was successful in eliminating any trace elements or PRQ or CPRQ.

### Accuracy and precision

Inter-assay precision, intra-assay precision and accuracy were within ± 14%, ± 10% and ± 6%, respectively, for ( +) and (−) of PRQ and CPRQ enantiomers (LLOQ, QC, ULOQ, and over-curve dilution) evaluated during four days of validation (Table 1). This met regulatory requirements of ± 15% for QC samples and ± 20% for LLOQ samples. The LLOQ and limit of detection (LOD) were set to 0.571 ng/mL and 0.286 ng/mL, respectively, for each enantiomer of PRQ and to 2.44 ng/mL and 1.22 ng/mL, respectively, for each enantiomer of CPRQ samples. This is a substantial improvement in the sensitivity of PRQ compared to the previously published method reporting LLOQ and LOD at 5 ng/mL and 2 ng/mL, respectively for PRQ [23]. Validation samples were prepared using EDTA plasma, but alternative anticoagulants (i.e., fluoro-oxalate and heparin) were evaluated by five replicates of QC samples prepared in fluoro-oxalate and heparin plasma. The overall mean accuracy was 101% and 99.0% for heparin and fluoro-oxalate, respectively, and the overall precision was 3.62% and 2.86%, respectively, for heparin and fluoro-oxalate samples. These results suggested that EDTA, heparin and fluoro-oxalate can all be used as anticoagulants with the validated method.

**Table 1:** Accuracy and precision for the quantification of ( ±)-primaquine and ( ±)-carboxyprimaquine in human EDTA plasma

Analyte	Sample	Nominal concentration (ng/mL)	Measured concentration (ng/mL)	Accuracy (%)	Between-assay precision (RSD)	Within-assay precision (RSD)
( +)-PRQ	LLOQ	0.571	0.591	103	7.72	9.93
	QC1	1.46	1.48	101	6.38	6.32
	QC2	16.8	16.8	100	6.27	3.60
	QC3	195	193	98.9	3.08	3.54
	ULOQ	260	256	98.5	6.77	4.00
	Over curve	519	515	99.2	4.20	2.81
(−)-PRQ	LLOQ	0.571	0.556	97.4	13.3	9.08
	QC1	1.46	1.43	97.9	7.12	4.54
	QC2	16.8	17.1	101	6.67	1.13
	QC3	195	192	98.5	1.45	1.03
	ULOQ	260	252	96.9	2.80	1.05
	Over curve	519	530	102	1.62	1.64
( +)-CPRQ	LLOQ	2.44	2.39	97.9	8.12	4.93
	QC1	7.32	7.28	99.5	8.84	9.13
	QC2	117	116	99.1	3.34	2.50
	QC3	1875	1806	96.3	7.61	2.19
	ULOQ	2500	2471	98.8	9.31	2.39
	Over curve	5000	4886	99.7	7.45	3.87
(−)-CPRQ	LLOQ	2.44	2.31	94.7	5.90	8.23
	QC1	7.32	7.22	98.6	11.2	7.45
	QC2	117	114	97.4	5.18	1.68
	QC3	1875	1813	96.7	4.70	4.03
	ULOQ	2500	2404	96.2	2.80	1.05
	Over curve	5000	4938	98.8	1.62	1.64

CPRQ, carboxyprimaquine; LLOQ, lower limit of quantification; PRQ, primaquine; QC, quality control; RSD, relative standard deviation; ULOQ, upper limit of quantification

### Stability

Short-term and long-term stability were evaluated for all procedures in the assay, including storage of clinical samples, sample preparation and LC–MS/MS processes. ( ±)-PRQ and ( ±)-CPRQ were stable in EDTA plasma during 3 freeze/thaw cycles, at ambient temperature for at least 48 h, and at 4 °C for at least 48 h. Precipitated samples were stable for at least 2 h at ambient temperature. Extracted ( ±)-PRQ and ( ±)-CPRQ, ready for injection, were stable for at least 60 h in the autosampler (4 °C). Stock solutions of ( ±)-PRQ and ( ±)-CPRQ were stable at ambient temperature for at least 4 h, 1 week in refrigerator (4 °C) and 1 month at − 80 °C. ( ±)-PRQ and ( ±)-CPRQ in EDTA plasma showed long-term stability for up to 2 years when stored at − 80 °C. During these conditions, concentrations of ( ±)-PRQ and ( ±)-CPRQ deviated less than 10% from their nominal concentrations. These stability data demonstrated that PRQ and CPRQ were stable during all procedures during routine performance of the method, and also during long-term storage of clinical study samples.

### Dilution integrity

The ULOQ was set to cover peak concentrations of ( ±)-PRQ and ( ±)-CPRQ expected after standard treatment. However, over-curve samples were evaluated at 2 × ULOQ to allow dilution and quantification of outliers. Five replicates of these samples were diluted five times with EDTA blank plasma, and showed an overall accuracy and precision of 97–102% and 0.52–7.79%, respectively. These results confirm that samples outside the calibration range can be diluted enabling such samples to be quantified reliably.

### Absolute recovery, matrix effects and selectivity

The absolute recovery of ( ±)-PRQ and ( ±)-CPRQ and their internal standards were in the range of 70–80% at all QC levels (Table 2). The absolute recoveries of analytes and isotope-labelled internal standard were highly correlated, producing a normalized recovery (analyte/internal standard) close to 1. This demonstrated that the internal standards compensated fully for any deviations in the recovery of the analytes. Similar recoveries have been reported in the literature when using liquid–liquid extraction (60–65%) [21], solid phase extraction (> 85%) [22] and direct protein precipitation (80–90%) [23]. The absolute recovery presented here was somewhat lower compared to direct protein precipitation and solid-phase extraction, but the phospholipid removal was necessary in order to enable a sensitive method without accumulation of phospholipid residues during high-volume clinical sample analysis.

**Table 2:** Absolute recovery, matrix effects and normalized matrix effects of ( ±)-primaquine, ( ±)-carboxyprimaquine and their isotope-labelled internal standard in human EDTA plasma

Analyte	Sample	R1	R2	R3	R4	R5	Average		SD﻿	CV (%)
Absolute recovery (%), (n = 5)										
( +)-PRQ	QC1	75.3	73.3	76.7	74.6	71.3	74.2		2.05	2.76
	QC3	72.3	78.4	72.3	70.9	70.5	72.9		3.19	4.38
	IS QC1	76.8	76.4	76.1	71.9	73.2	74.9		2.19	2.92
	IS QC3	74.0	78.4	73.7	72.5	71.1	73.9		2.74	3.71
(−)-PRQ	QC1	78.1	72.3	73.1	71.0	75.3	74.0		2.79	3.78
	QC3	76.8	70.7	75.7	74.9	72.3	74.1		2.51	3.39
	IS QC1	73.3	73.4	73.3	75.3	70.5	73.2		1.71	2.34
	IS QC3	78.2	71.4	75.9	75.3	73.6	74.9		2.55	3.40
( +)-CPRQ	QC1	75.1	75.1	79.2	73.0	72.3	74.9		2.69	3.59
	QC3	75.2	75.2	78.0	74.7	75.4	75.7		1.31	1.73
	IS QC1	77.6	77.6	80.1	75.2	77.8	77.7		1.73	2.23
	IS QC3	73.5	73.5	74.6	72.9	73.1	73.5		0.66	0.89
(−)-CPRQ	QC1	79.2	79.8	74.1	76.8	70.5	76.1		3.85	5.05
	QC3	76.5	78.9	75.5	76.7	73.4	76.2		2.00	2.62
	IS QC1	73.7	76.3	70.4	72.9	73.2	73.3		2.11	2.87
	IS QC3	75.4	78.2	74.6	75.2	73.8	75.4		1.66	2.21

Analyte	Sample	R1	R2	R3	R4	R5	R	Average	SD	CV (%)
Matrix effects, (n = 6)										
( +)-PRQ	QC1	1.08	1.05	1.14	1.15	1.20	1.03	1.11	0.07	5.91
	QC3	1.01	0.91	1.03	1.00	0.96	1.12	1.01	0.07	7.03
	IS QC1	0.98	0.97	0.98	1.13	1.04	1.00	1.02	0.06	5.99
	IS QC3	0.91	1.04	0.93	0.94	0.85	0.97	0.94	0.06	6.73
(−)-PRQ	QC1	1.14	1.10	1.08	1.15	1.14	1.09	1.12	0.03	2.70
	QC3	0.95	1.08	1.06	1.20	1.06	1.04	1.07	0.08	7.55
	IS QC1	1.09	1.11	1.09	1.07	0.98	1.10	1.07	0.05	4.44
	IS QC3	1.06	1.01	1.02	1.09	1.12	1.02	1.05	0.04	4.23
( +)-CPRQ	QC1	0.83	0.89	1.03	1.06	0.92	0.96	0.95	0.09	9.13
	QC3	1.10	1.20	1.17	1.08	1.04	1.06	1.11	0.06	5.72
	IS QC1	0.94	1.10	1.05	0.94	0.92	0.98	0.99	0.07	7.33
	IS QC3	1.06	1.14	1.22	1.12	1.04	1.17	1.13	0.07	6.00
(−)-CPRQ	QC1	0.99	1.02	1.09	0.93	0.95	1.04	1.00	0.06	5.90
	QC3	1.10	1.15	1.14	1.03	1.08	1.06	1.09	0.05	4.24
	IS QC1	1.08	1.09	1.08	1.09	1.02	1.03	1.07	0.03	2.95
	IS QC3	1.02	1.19	1.05	0.99	0.99	1.01	1.04	0.08	7.30
Normalised matrix effects, (n = 6)										
( +)-PRQ	QC1/IS QC1	1.10	1.08	1.16	1.02	1.15	1.03	1.09	0.06	5.57
	QC3/IS QC3	1.11	0.88	1.11	1.06	1.13	1.15	1.07	0.10	9.47
(−)-PRQ	QC1/IS QC1	1.05	0.99	0.99	1.07	1.16	0.99	1.04	0.07	6.59
	QC3/IS QC3	0.90	1.07	1.04	1.10	0.95	1.02	1.01	0.08	7.62
( +)-CPRQ	QC1/IS QC1	0.88	0.81	0.98	1.13	1.00	0.98	0.96	0.11	11.5
	QC3/IS QC3	1.04	1.05	0.96	0.96	1.00	0.91	0.99	0.05	5.54
(−)-CPRQ	QC1/IS QC1	0.92	0.94	1.01	0.85	0.93	1.01	0.94	0.06	6.33
	QC3/IS QC3	1.08	0.97	1.09	1.04	1.09	1.05	1.05	0.05	4.42

CPRQ, carboxyprimaquine; CV, coefficient of variation; IS, Internal standard; PRQ, primaquine; QC, quality control; R, Replicate; SD, standard deviation

**Fig. 3:**
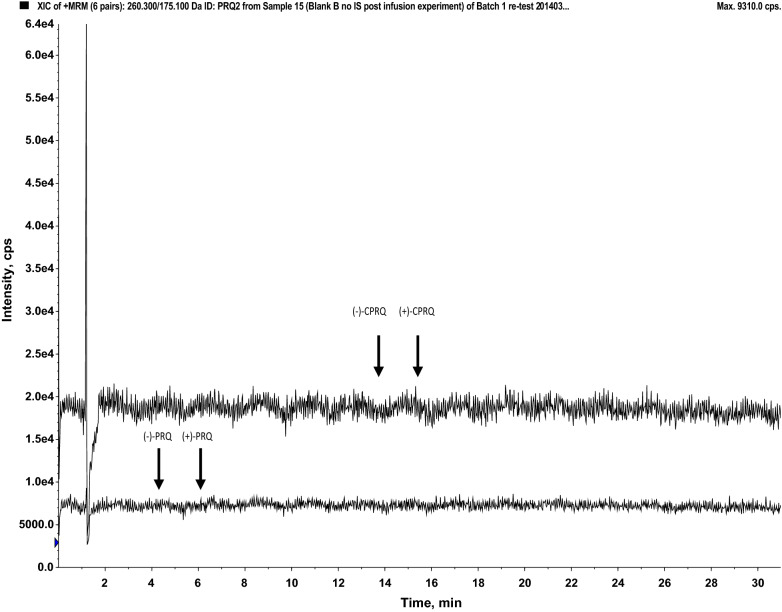
Visual evaluation of potential matrix effects on Chiralcel OD-3R column (150 mm × 4.6 mm; I.D. 3 μm) at flow rate 1.0 mL/min. Injection of extracted blank human plasma (volunteer A) during post column infusion (10 μL/min) of primaquine (25 ng/mL) and carboxyprimaquine (50 ng/mL). The lower chromatogram represents the extracted ion chromatogram (EIC) of PRQ and upper chromatogram represents the extracted ion chromatogram of CPRQ. The arrows indicate the retention times for ( ±)-primaquine and ( ±)-carboxyprimaquine

Post-column infusion (Fig. 3) and matrix effect calculations (Table 2) in different sources of plasma demonstrated that this method was free from any substantial matrix effect. The calculated matrix factor and matrix effect was within 0.85–1.15, and the relative matrix effect (% CV) was < 15% indicating no significant effects on the precision and accuracy of the assay. The normalized matrix factor was close to 1 with low variation, indicating that the internal standards compensated fully for any potential matrix effects. This was supported further by the qualitative matrix effect evaluation using post column infusion, demonstrating no visible ion suppression or enhancement at the retention times of the enantiomers of PRQ and CPRQ and internal standards (Fig. 3).

Specificity and selectivity were studied using 6 independent plasma samples from 6 different healthy volunteers. Post column infusion did not show any signs of ion suppression/enhancement or significant interference at the retention times of the enantiomers of PRQ and CPRQ, and internal standards. None of the blank sources produced a signal > 20% of LLOQ, demonstrating a highly selective method with a minimal risk of interference from different patient samples.

There was no interference by potentially co-administered anti-malarial drugs (i.e. piperaquine, dihydroartemisinin, chloroquine and pyronaridine) and should not impact the quantification of PRQ and CPRQ.

### Partial validation of racemic method

Enantiomeric separation and quantification might not always be necessary to answer a clinical question, and thus a simplified method for racemic separation and quantification was developed and validated.

**Fig. 4:**
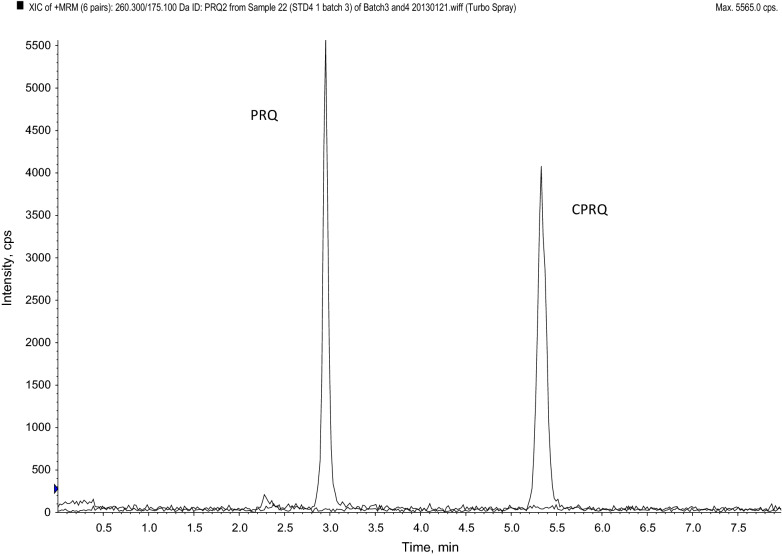
Retention time of racemic primaquine and carboxyprimaquine on the Hypersil Gold column (100 mm × 4.6 mm; I.D. 3 μm) at a flow rate of 0.5 mL/min, using an isocratic mobile phase (10 mM ammonium acetate pH 3.5:acetonitrile; 50:50, v/v)

**Fig. 5:**
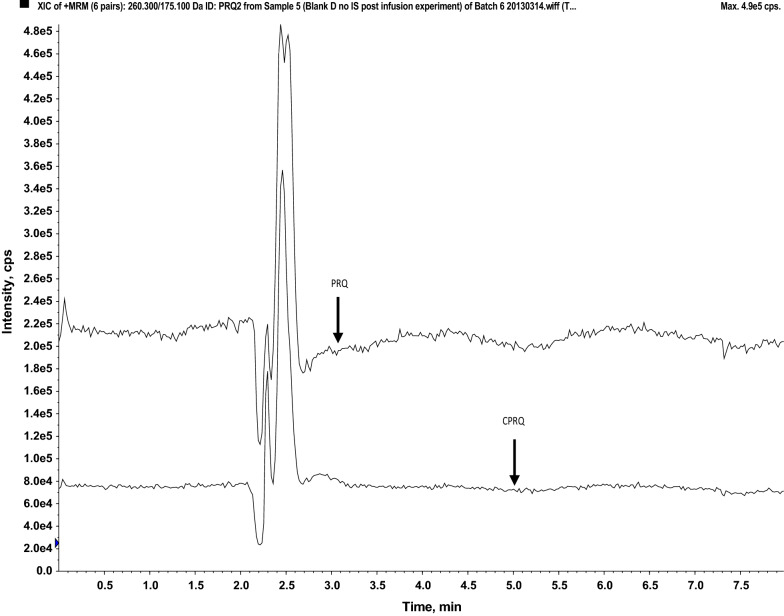
Visual evaluation of potential matrix effects on the Hypersil Gold column (100 mm × 4.6 mm; I.D. 3 μm) at a flow rate of 0.5 mL/min. Injection of extracted blank human plasma (volunteer A) during post-column infusion (10 μL/min) of primaquine (10 ng/mL) and carboxyprimaquine (20 ng/mL). The upper chromatogram represents the extracted ion chromatogram (EIC) of PRQ and lower chromatogram represents the extracted ion chromatogram of CPRQ. The arrows indicate the retention times for primaquine and carboxyprimaquine

A number of different C18 columns (Hypersil Gold C18, Gemini C18) and CN columns (Hypersil CN) were evaluated for optimal separation of racemic PRQ and CPRQ. All columns achieved acceptable separation using an isocratic mobile phase at varying concentrations of ammonium acetate and acetonitrile. However, the best peak symmetry was achieved with the Hypersil Gold column (100 mm × 4.6 mm; I.D. 3 μm) and an isocratic mobile phase (10 mM ammonium acetate pH 3.5:acetronitrile, 50:50, v/v) resulting in a total run time of 8 min (Fig. 4). PRQ, PRQ-D3, CPRQ and CPRQ-D3 eluted at 2.95 min, 3.00 min, 5.33 min and 5.39 min, respectively. Detection of PRQ, CPRQ and isotope-labelled internal standards was performed using the following MRM transitions m/z 260.3 > 175.1 (PRQ), m/z 263.3 > 86.1 (PRQ-D3), m/z 275.2 > 175.1 (CPRQ) and m/z 278.4 > 178.1 (CPRQ-D3). The MS parameters were identical to that described for the enantiomeric separation method, except at Q_0_ (front end). The front end was optimized with respect to ion source gas (GS1; 50 psi) and temperature (450 °C) for optimum results for PRQ and CPRQ. The developed method showed no signs of interference (i.e. ion suppression/enhancement) at the retention times of PRQ, CPRQ and internal standards (Fig. 5). The internal standards compensated satisfactorily for any deviations and the normalized matrix factors were close to 1 with low variation (Table 3). The calibration range was set to 1.14–519 ng/mL and 4.88–5,000 ng/mL for PRQ and CPRQ, respectively. The overall accuracy, within-day precision and between-day precision were below 5% at all quality control samples of PRQ and CPRQ in human plasma using the developed racemic method.

**Table 3:** Matrix effect and normalised matrix factor for racemic primaquine and carboxyprimaquine

Analyte	Sample	R1	R2	R3	R4	R5	R6	Average	SD	CV (%)
Matrix effects (%), (n = 6)										
PRQ	QC1	1.17	1.22	1.07	1.10	1.10	1.11	1.13	0.06	4.93
	QC3	0.92	0.88	0.97	0.99	1.03	0.95	0.96	0.05	5.43
	IS QC1	1.04	1.11	0.95	1.03	0.96	1.02	1.02	0.06	5.74
	IS QC3	0.95	0.90	0.98	1.01	1.05	0.97	0.98	0.05	5.19
CPRQ	QC1	0.99	1.07	1.02	0.98	0.96	0.97	1.00	0.04	4.08
	QC3	1.03	0.98	0.98	1.05	1.00	1.00	1.01	0.03	2.73
	IS QC1	1.02	1.07	0.97	0.93	0.99	0.96	0.99	0.05	5.09
	IS QC3	1.08	1.06	1.16	0.98	0.98	0.98	1.04	0.07	7.09
Normalised matrix effect (n = 6)										
PRQ	QC1/IS QC1	1.13	1.10	1.13	1.07	1.15	1.09	1.11	0.03	2.59
	QC3/IS QC3	0.97	0.98	0.98	0.98	0.98	0.99	0.98	0.01	0.60
CPRQ	QC1/IS QC1	0.97	1.00	1.05	1.06	0.97	1.01	1.01	0.04	3.78
	QC3/IS QC3	0.95	0.93	0.85	1.07	1.02	1.02	0.97	0.08	8.32

CPRQ, carboxyprimaquine; CV, coefficient of variation; IS, Internal standard; PRQ, primaquine; QC, quality control; R, Replicate; SD, standard deviation

Most racemic methods for quantification of PRQ and CPRQ have used UV-detection [12, 16, 44], but a recently published method reported a LC–MS/MS method [22]. The latter method requires 500 µL of plasma and a total run time of 11 min, with a LLOQ at 2.0 and 2.5 ng/mL for PRQ and CPRQ, respectively [22]. The validated racemic method, reported here, showed a slightly increased sensitivity for PRQ (LLOQ of 1.14 ng/mL) and a shorter runtime of 8 min, but with the main advantage of requiring only 100 µL of plasma.

## Clinical applicability

### Enantiomeric separation method

**Fig. 6:**
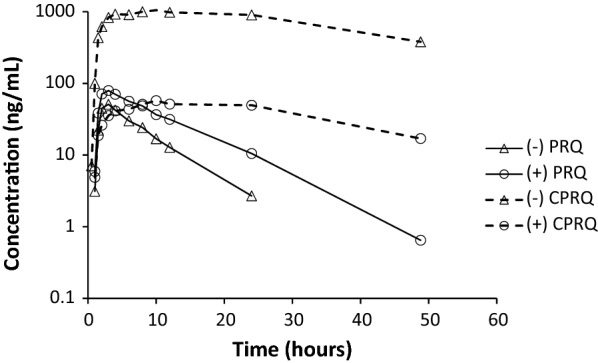
Measured plasma concentration–time profiles of (−)-PRQ, ( +)-PRQ, (−)-CPRQ and ( +)-CPRQ in one healthy volunteer after administration of a single oral dose of 30 mg primaquine phosphate

Two drug-drug interaction studies in healthy volunteers in Thailand [34, 35] were analysed using the described enantiomeric separation LC–MS/MS method. The accuracy and relative standard deviation (RSD) were below 10% at all QCs levels. The resulting plasma concentration–time profiles of each enantiomer of PRQ and CPRQ from a healthy volunteer is shown in Fig. 6. The concentration range of the clinical samples was fully covered by the enantiomeric method and none of the clinical samples required reanalysis with dilution, demonstrating the appropriateness of the developed method. The maximum concentration of ( +)-PRQ was approximately twice that for (-)-PRQ, while there was a more than tenfold difference in the maximum concentrations of ( +)-CPRQ and (-)-CPRQ. These enantiospecific pharmacokinetic properties are similar to those reported previously in healthy volunteers [15, 34, 35]. Data from the two studies above were also pooled and evaluated using a population pharmacokinetic modelling approach [45]. This pooled analysis in 49 healthy adult volunteers demonstrated a 2.5-fold increase in the exposure to plasma ( +)-PRQ compared with (−)-PRQ. Additionally, there was an even larger difference in the exposure to the carboxyprimaquine enantiomers, resulting in a 21-fold higher exposure to (−)-CPRQ compared with ( +)-CPRQ. These large pharmacokinetic differences in enantiomers suggests that further evaluations are needed urgently. Radical cure of *P. vivax* malaria and adverse events, such as gastrointestinal disturbance and haemolytic toxicity, might be improved by characterizing the toxicokinetic profiles of primaquine enantiomers in patients, particularly in individuals with mild to moderate G6PD deficiency. The presented LC–MS/MS method could thus be applied to such as study in measuring plasma enantiomeric primaquine and carboxyprimaquine concentrations. In support of this, Saunders et al. showed pharmacokinetic and pharmacodynamic differences of the enantiomers of primaquine administered to *P. cynomolgi*-infected rhesus macaques and recommended further investigations to evaluate potential toxicokinetic advantages of the enantiomers [9].

The reliability of the validated method was confirmed by reanalysis of clinical samples (i.e. incurred sample reanalysis, ISR). Reanalysis was performed on 83 randomly selected samples above the LLOQ, out of 800 clinical samples analysed in total (10% of the total number of samples). Only 4 PRQ and 4 CPRQ samples out of 83 samples (4.8%) showed a deviation above 20% (21.3–28.0) compared to the original analysis value, which is well within the regulatory guideline for ISR.

### Racemic method

A drug-drug interaction study of primaquine and chloroquine in healthy volunteers in Thailand [33] were analysed using the newly-developed racemic method. All of the plasma samples were within the calibration range and none of the clinical samples required reanalysis with dilution, demonstrating the appropriateness of the developed method. The accuracy and RSD were below 7% at all QCs levels. There was an approximate tenfold difference in the maximum concentrations of PRQ and CPRQ, as previously reported [12, 16, 43, 46].

The reliability of the validated method was confirmed by reanalysis of clinical samples. Reanalysis was performed on 73 randomly selected samples above the LLOQ, out of 528 clinical samples analysed in total (14% of the total number of samples). None of the 73 samples showed a deviation above 20% compared to the original analysis value.

## Conclusions

The newly developed enantiomeric and racemic separation and detection methods for PRQ and CPRQ proved sensitive, accurate, precise, and reproducible. The sensitivity (LLOQ) was 0.571 and 2.44 ng/mL for each enantiomer of PRQ and CPRQ, respectively, while the racemic method showed a sensitivity of 1.14 and 4.88 ng/mL for PRQ and CPRQ, respectively. Protein precipitation followed by phospholipid removal SPE showed an excellent recovery with no interference from co-administered drugs or matrix effects. The developed and validated methods are more sensitive than previously published methods, with the advantage of requiring substantially smaller samples volume of 100 µL and relatively short sample runtime of 30 and 8 min for the enantiomeric and racemic method, respectively. The presented methods were implemented successfully in high-throughput routine analysis of pharmacokinetic clinical trial samples.

## Data Availability

All data generated or analysed during this study are included in this published article and its additional files.

## Abbreviations

PRQ: Primaquine
CPRQ: Carboxyprimaquine
PRQ-D3: Primaquine-deuterium3
CPRQ-D3: Carboxyprimaquine-deuterium3
LC–MS/MS: Liquid chromatography-tandem mass spectrometry
LLOQ: Lower limit of quantification
ULOQ: Upper limit of quantification
MRM: Multiple Reaction Monitoring
